# Omics Data and Their Integrative Analysis to Support Stratified Medicine in Neurodegenerative Diseases

**DOI:** 10.3390/ijms22094820

**Published:** 2021-05-01

**Authors:** Valentina La Cognata, Giovanna Morello, Sebastiano Cavallaro

**Affiliations:** Institute for Biomedical Research and Innovation (IRIB), Italian National Research Council (CNR), 95126 Catania, Italy; valentina.lacognata@cnr.it (V.L.C.); giovanna.morello@irib.cnr.it (G.M.)

**Keywords:** neurodegenerative diseases, multi-omics, stratified medicine

## Abstract

Molecular and clinical heterogeneity is increasingly recognized as a common characteristic of neurodegenerative diseases (NDs), such as Alzheimer’s disease, Parkinson’s disease and amyotrophic lateral sclerosis. This heterogeneity makes difficult the development of early diagnosis and effective treatment approaches, as well as the design and testing of new drugs. As such, the stratification of patients into meaningful disease subgroups, with clinical and biological relevance, may improve disease management and the development of effective treatments. To this end, omics technologies—such as genomics, transcriptomics, proteomics and metabolomics—are contributing to offer a more comprehensive view of molecular pathways underlying the development of NDs, helping to differentiate subtypes of patients based on their specific molecular signatures. In this article, we discuss how omics technologies and their integration have provided new insights into the molecular heterogeneity underlying the most prevalent NDs, aiding to define early diagnosis and progression markers as well as therapeutic targets that can translate into stratified treatment approaches, bringing us closer to the goal of personalized medicine in neurology.

## 1. Introduction

Neurodegenerative diseases (NDs) are debilitating and largely untreatable conditions characterized by a decline of nervous system functions due to a progressive neuronal loss in the brain and spinal cord. The classification of NDs is still usually based on the clinical presentation (i.e., cognitive decline, speech difficulties and motor impairment), anatomical regions and cell types affected [1,2]. As the exact molecular mechanisms of the disease pathogenesis and progression remain unclear, the clinical management of NDs is limited to simply mitigating neurodegeneration and relieving symptoms rather than reversing the damage done [1,3,4].

NDs can be either monogenic, like Huntington disease, or complex, highly heterogeneous—including Alzheimer’s disease (AD), Parkinson’s disease (PD) and amyotrophic lateral sclerosis (ALS)— and characterized by variable molecular phenotypes, progression courses or patterns of neuro-biochemical markers of brain damage, making patient counseling, disease management and pharmaceutical care particularly difficult [3]. The underlying mechanisms of these complex NDs are polyfactorial and depend on the combination of genetic, biological and environmental factors. The presence of abnormal protein conformations, excessive immune response and inflammation, impaired nucleocytoplasmic transport, mitochondrial dysfunction, neuronal dysfunction and autophagy are common features of neurodegeneration [5,6]. However, despite considerable efforts, the molecular mechanisms involved in the complex phenotype of NDs are still largely unknown, and current treatments cannot prevent the development of the disease. The failure of the majority of neurological clinical trials, especially during Phase 3, can be attributed to a lack of efficacy, probably due to the incorrect selection of the target population [7,8].

To ensure a more accurate diagnosis and design more appropriate clinical trials, we need to decipher molecular signatures, pathways and networks that can specifically characterize different disease subtypes for the correct classification of patients. In this context, personalized or at least stratified medicine for patients’ subgroups offers the possibility of repurposing disease-modifying drugs or identifying new potential medical solutions to ensure “*the right therapeutic strategy for the right person at the right time*” [9,10].

**Figure 1:**
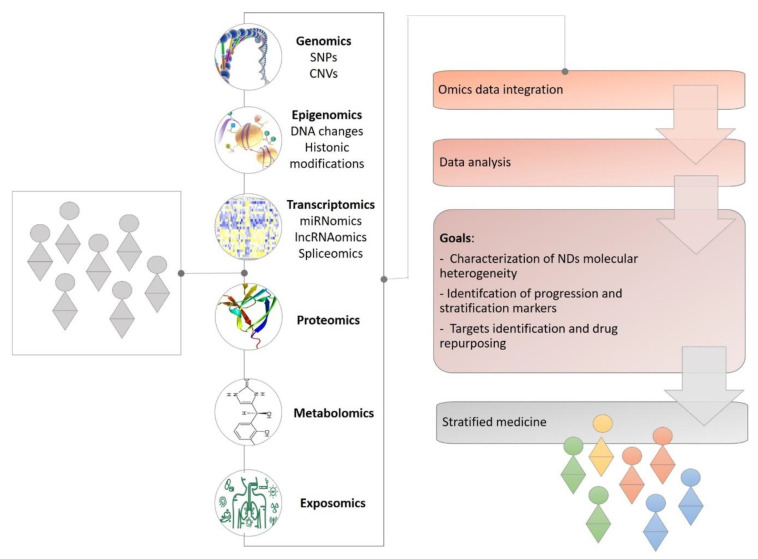
A full readout of ND conditions to support stratified medicine. From the genome onwards, information gathered from all omics molecular layers of NDs conditions will aid researchers and clinicians to better characterize the disease’s molecular heterogeneity, stratify patients by novel biomarkers and improve therapeutic outcomes.

In the last years, the advance of high-throughput “omics” techniques has provided a more complete view with respect to the complexity of NDs from multiple levels (e.g., network, cellular and molecular), encouraging the identification of specific molecular signatures and biomarkers for mechanism-based classification and tailored therapeutic interventions [11,12]. These technologies include the detection of disease-associated DNA sequence variants (genomics), transcriptome and noncoding RNA profiling (transcriptomics), genome-wide identification of DNA–protein interactions (epigenomics), interactome analysis for networks formed by protein-protein interactions (proteomics) and metabolome analysis for metabolic systems (metabolomics) [13] (Figure 1).

However, single “omics” analyses, by capturing changes only for a small subset of the components of a particular pathway, have limited prognostic or therapeutic value. The majority of human diseases, including NDs, are multifactorial and characterized by a plethora of molecular aberrations that act in a concurrent or synergistic way during the development of the disease. Therefore, the analysis and integration of all these biological big data allow for the simultaneous identification of molecular aberrations at different levels (gene, transcript, protein synthesis and post-translational modifications, cellular metabolic processes, etc.), maximizing the available information, and thus increasing the possibility of identifying the root causes of the disease. In fact, individual changes in gene expression or protein, metabolite and lipid concentration may have limited translational potential, but when combined they increase the possibility of a particular gene or protein and related pathways to play a crucial role in the disease’s pathogenesis. The multi-omics analyses and the characterization of an “omic” profile of patients have started to enable a deeper investigation of NDs providing a more comprehensive overview of these complex and multifactorial disorders, promoting the development of patient-specific precision-targeted personalized therapies to effectively treat neurodegenerative disorders [14,15,16] (Figure 1).

In this review, we provide an overview of the current state of the field and how the progress made in genomics, transcriptomics, proteomics, epigenomics and metabolomics is offering a new perspective to uncover the molecular heterogeneity underlying the most prevalent NDs, aiding to refine early diagnosis, depict patient subgroups, guiding the development of therapies and improving drug discovery efforts. In particular, the literature consulted for this review includes original research contributions, academic and perspective articles published over the last five years and focused on the use of omics science for disease taxonomy and patient subtyping. In addition, where possible, we also discuss how bringing data from these techniques together through an integrated “systems biology” view will move our understanding and management of NDs forward, bringing us closer to the goal of stratified medicine in neurology.

## 2. Alzheimer’s Disease

Alzheimer’s disease (AD) is the most common form of brain dementia, affecting over 44 million individuals worldwide and clinically characterized by a progressive loss of memory, cognitive decline and neurodegeneration [17]. In general, the physiopathology of AD includes a loss of synapses, mainly related to the accumulation of the β-amyloid peptide into extracellular plaques and intraneuronal aggregates of the abnormally hyperphosphorylated microtubule-associated protein tau, particularly in the hippocampus and neocortex. These events induce generalized neuroinflammation, vascular and cell membrane dysregulation, axonal disintegration and synaptic dysfunction and degeneration, brain metabolic dysfunction and, ultimately, a deterioration of physiological neural connectivity [18]. Currently, the diagnosis of AD is confirmed through post-mortem analysis and the identification of the neurofibrillary tangles and/or abnormal plaque deposits within the brain. Only approximately <5% of the AD cases are familial forms of autosomal dominant inheritance and are generally characterized by an early-onset and associated to genetic mutations in some proteins (e.g., presenilin 1, presenilin 2, amyloid-*β* protein precursor A*β*PP), while 95% cases of AD are late-onset and sporadic, resulting from a complex interaction of genes and environmental factors [19].

During the last ~25 years, an impressive amount of progress has been made in the understanding of the genetic causes and molecular mechanisms related to AD. Recent advances in AD genomics and high-throughput sequencing, as well as large-scale genome-wide association studies (GWAS), allowed us to investigate not only the principal disease-causative genes but also several low-frequency genetic loci that seem to exert large effects on AD risk. These studies highlighted a significant locus heterogeneity for AD and indicated that common variants with small effect sizes in combination with many rare genetic variants with moderate to large effect sizes may jointly contribute to AD risk [20]. Single-cell RNA-sequencing analyses allowed for the identification of a novel microglia type associated with AD and other neurodegenerative diseases (known as disease-associated microglia, DAM), whose genetic and functional characterization can be used to evaluate the preclinical feasibility of new, more promising drug targets [21]. Moreover, a distinctive blood-based transcriptomic signature of AD emerged when compared to other neurological diseases, favoring the development of a blood RNA test (e.g., AclarusDx™) able to discriminate rapidly progressing AD patients and slowly progressing patients with other forms of dementia before the onset of the disease [22,23].

In addition to the single-omic analysis, results from multiple multi-omic analyses have greatly advanced the understanding of AD pathogenesis not only by revealing its global structures, but also by detailing circuits of complex molecular interactions and regulations in affected key brain regions (Table 1) [24,25,26,27,28]. In Nativio et al. (2020), for example, the authors described the utility of an integrated transcriptomic, proteomic and epigenomic approach of postmortem human brains to identify the molecular pathways involved in AD [28], while Xicota et al. (2019) performed an integrative blood RNAseq, and plasma metabolomics and lipidomics, by generating a blood omics signature for the prediction of amyloid positivity in asymptomatic at-risk subjects, allowing for a less invasive, more accessible and less expensive risk assessment of AD [27] (Table 1). A recent study using deep profiling of whole proteome, phosphoproteome and transcriptome in different disease stages of AD illustrates the ability of deep proteomics technologies to complement genomics and transcriptomics in AD research, revealing crucial molecular networks and pathways associated with AD and demonstrating that protein changes are only partially consistent with the corresponding RNA levels [29]. Taken together, these and other multi-omic studies, thanks also to the implementation of publicly available datasets in international projects such as the Alzheimer’s Disease Neuroimaging Initiative (ADNI) and the Religious Orders Study and Memory and Aging Project (ROSMAP), provide a valuable resource for more comprehensive analyses of AD, representing a potential key enabler of novel biomarker discovery [30,31].

Despite such advancements in the understanding of the disease, establishing a definitive diagnosis and developing rational treatments is complicated, also because AD drug trials do not account for the heterogeneity of the disease in trial design [48]. In fact, it is widely recognized that AD is characterized by an etiological and clinical heterogeneity with substantial variability from patient to patient with respect to age at onset, disease manifestation, progression, response to treatment and susceptibility to risk factors and their downstream pathophysiologic consequences [49].

The substantial heterogeneity of AD from a genetic point of view emerged, for example, in recent genome-wide association studies revealing how the ∊4 allele of the apolipoprotein E (APOE) gene shows a dose-dependent relationship with increased risk of late-onset and sporadic cases of AD, while the inheritance of the ∊2 allele is protective, highlighting the possibility of stratifying AD patients based on their APOE genotype [32,50,51,52] (Table 1).

In another study, researchers categorized people with late-onset AD into six biologically coherent subgroups based on clinical symptomatology and genetic backgrounds [53]. Additional stratification analyses of AD patients were also obtained by analyzing whole genome sequencing and whole transcriptome data from post-mortem brain tissues of AD patients, identifying clusters of patient-specific transcriptional signatures and demonstrating the high molecular variability and complexity of gene expression in AD [33,34,35] (Table 1). In particular, Neff et al. (2021), by analyzing transcriptomes across different AD affected brain regions, identified three major molecular subtypes of AD independent of age and disease severity, each one characterized by different combinations of dysregulated pathways and a unique set of key regulator genes, suggesting that specific gene modules are subtype-specific, and subtypes may be driven by a specific, yet diverse set of disease mechanisms that lead to AD [35].

In view of the translation of molecular-based stratification into clinical practice, cerebrospinal fluid (CSF) biomarker-guided stratification is proving helpful to identify specific AD patient subgroups and can also serve as the outcome measure of drug treatment. To this end, recent studies have demonstrated the possibility to subdivide AD patients into different clusters based on the CSF levels of a set of potential biomarkers (including Aβ1−42, tau and ubiquitin), demonstrating that each cluster was associated with a different clinical profile and thus potentially different disease-related trajectories and drug responses [36,37,54] (Table 1).

In addition to genomics, transcriptomics, proteomics or other omics (i.e., epigenomics and metabolomics) are moving toward a better definition and characterization of AD heterogeneity. Recently, Nazarian et al. (2020) performed methylome-wide association analyses of blood and brain tissue samples from AD patients and identified group-specific methylation quantitative trait loci, suggesting a potential role for such epigenetic modifications in the heterogeneous nature of AD [55]. Similarly, the evidence that APOE, a lipid chaperone protein, is the most important genetic risk factor for sporadic late-onset of AD, suggests the importance of exploring lipid and metabolic dynamics in AD research. To this end, several international projects (e.g., HUSERMET and PredictAD) are currently available and aim to define serum-derived metabolic markers in AD [56]. Results from these and other metabolomics studies have allowed us to assess multiple combinations of metabolites to discriminate different AD subtypes characterized by different molecular mechanisms and clinical manifestations, advancing efforts to biochemically define patient heterogeneity in AD [57,58,59,60,61].

Another interesting aspect of multi-omics-based advances is the possibility of applying these methodologies to accelerate target identification for drug discovery in AD. In this regard, network-based drug repurposing offers a rapid and cost-effective solution for drug discovery for complex diseases, like AD, and some studies have started to use this approach for the discovery of drugs that show efficacy in network models in AD (e.g., sildenafil, pioglitazone), providing potential mechanisms for these drugs and facilitating their subsequent experimental validation [22,23]. Recently, a new freely available database and tool, termed AlzGPS (https://alzgps.lerner.ccf.org, accessed 30 April 2021), was developed, containing rich and diverse information connecting large-scale data, including multi-omics (genomics, transcriptomics, proteomics, metabolomics, interactomics) on humans and other species, literature-derived evidence, drug-target networks, clinical databases for genome-informed target identification and drug repurposing for AD [24]. Taking advantage of this and other computational platforms, it will be possible to prioritize biologically and clinically relevant targets and relative drug candidates for multi-omics-informed discovery in AD and other neurodegenerative diseases.

## 3. Parkinson’s Disease

Parkinson’s disease (PD) is a chronic debilitating and still incurable neurodegenerative disorder, characterized by a variable combination of motor and non-motor symptoms, a heterogeneous rate of disease progression and different prognostic outcomes [62]. It increases with age, affecting more than 1% of the population over 60 years of age, with a worldwide incidence rate of 8–18 per 100,000 person-years. From a neuropathological point of view, PD is characterized by a selective degeneration of dopaminergic neurons in the pars compacta region of the substantia nigra (SNc) and by the cytoplasmic and axonal accumulation of aggregated misfolded α-synuclein into Lewy bodies (LB) and Lewy neurites (LN) [63].

Although the precise etiology of this disease remains largely unclear, multiple genetic and environmental factors have been elucidated during the last decades. About 5–10% of all patients suffer from a monogenic form of PD where mutations in autosomal-dominant (AD) genes—SNCA, LRRK2 and VPS35—and autosomal recessive (AR) genes—PINK1, DJ-1 and PARK2—cause the disease [63]. The vast majority of PD corresponds to complex multifactorial sporadic cases without a family history, resulting from a combination of common genetic risk loci in concert with environmental factors (lifestyle, exposure to toxins, physical activity), and triggered by several molecular processes (e.g., synaptic damages, apoptosis, mitochondrial dysfunctions, oxidative stress, impairment of the ubiquitin/proteasome system, neuro-inflammation) [64].

The large heterogeneity of PD in clinical presentations, together with the presence of different genetic or environmental causes, has currently led to the suggestion that what we term PD is actually a collection of distinct disease entities [62]. The current traditional PD patients classification is mainly based on clinical disease milestones such as age at disease onset (juvenile, early and typical forms), demographic profiles, motor phenotypes (tremor-dominant and non-tremor-dominant), the severity of motor symptoms based on functional scores (hypo-/bradykinesia, rigidity, rest tremor, postural instability—Unified Parkinson Disease Rating Scale) and neuropathological alterations (Braak staging) [65]. Moreover, non-motor features (cognitive performance indicators, apathy, depression/anxiety, dementia status and the co-occurrence of REM sleep behavior disorder) are increasingly receiving major attention, since they can precede the diagnosis for years and can help to prognosticate disease progression [65].

Despite these attempts to stratify patients into the disease-related motor or non-motor clinical patterns [66,67], classifications fall short of comprehensively describing and characterizing the broad, continuous and multidimensional spectrum of PD manifestations and their progression under real-life conditions. Therefore, these initial empirical classifications are starting to be gradually replaced by data-driven cluster analyses without a priori hypotheses [68,69,70,71] or integrative multi-level studies combining detailed clinical information, omics information and neuropathological findings [72]. However, the search for more refined taxonomic systems is still ongoing, in order to facilitate a better clinical care and personalized therapeutic decisions.

Firstly, patients’ stratification based on genetic status is turning out to be a particularly helpful approach to certain subtypes of PD cases in order to address clinical trials and therapeutic targeted pilot studies. For example, (i) LRRK2 inhibitors (DNL201 and DNL151) were specifically designed by Denali Therapeutics with the aim of restoring the LRRK-mediated lysosomal dysfunction in PD and have recently finished a double-blinded, placebo-controlled phase Ib drug trial [73]; (ii) a number of specific clinical trials focusing on PD patients carrying GBA mutations are underway (i.e., Venglustat—GZ/SAR402671 in NCT02906020; Ambroxol in NCT02914366) [74]; (iii) polymorphisms in the dopamine D2 receptor gene are under study for their meaningful predictive clinical response to rasagiline treatment [75], (iv) homozygous carriers of certain SNCA mutations are candidates for the positive outcome to deep-brain-stimulation [76]. However, this genetic stratification relying on causative Mendelian PARK gene mutations is unlikely to be applicable to the majority of sporadic PD cases.

To add further complexity, patients carrying mutations in the same gene locus (e.g., GBA) often manifest distinct phenotypic profiles, making it necessary to cluster patients into single-gene-related subgroups based on the variant types or into biochemical profiles for developing better disease-modifying strategies [77]. To this end, a metabolomics-based study investigating the biochemical metabolic profiles associated with GBA mutations (lysosomal GCase activity, glucosylceramides, ceramides, lactosylceramides, sphingosines, sphingomyelin and α-synuclein levels) in biofluids derived from PD patients carrying GBA mutations compared to PD-GBA-wildtype has recently confirmed that GBA variants have a relevant functional impact on biomarker profiles in patients, bridging the gap between genetics and biochemical status to allow an appropriate patient stratification for clinical trials [38] (Table 1).

A further interesting aspect raised by omics observations derives from a challenging clinical trial recently launched with the aim to assess the treatment response of the coenzyme Q10 (a “mitochondrial enhancer”) in four PD patient subgroups genetically stratified through an omics-score predictive for their potential “mitochondrial risk burden” (i.e., homozygous or compound heterozygous Parkin/PINK1 mutation carriers, heterozygous Parkin/PINK1 mutation carriers, “omics” positive and “omics” negative patients) [39] (Table 1). In this study, the authors aim to integrate data from these stratified groups about motor and non-motor symptoms, magnetic resonance imaging and changes in structural and functional brain anatomy (MRI). Clinical trial results are expected to provide cues about the utility of this omics-score to stratify PD patients as well as to provide findings about the opportunity to personalize treatment choices for PD based on the genetic, clinical and neuroimaging data [39].

PD precision medicine focusing on mechanistically-anchored disease subgroups derived from integrated omics findings may also hold promise [78]. In this regard, in-depth phenotyping of peripheral tissues from sporadic PD patients through a combination of cellular assays and whole-transcriptome RNA-seq based pathway analysis, along with genotyping information, allowed for the stratification of patients characterized by mitochondrial (mito-sPD) or lysosomal (lyso-sPD) main dysfunctions and facilitated the selection of putative neuroprotective compounds [40] (Table 1). This successful strategy of combining deep clinical phenotyping with a comprehensive assessment of genetic, transcriptomic and biological data, along with a focused assessment of putative neuroprotective compounds, is a promising approach toward disease stratification and precision medicine in sporadic PD.

Another example is represented by the Luxembourg Parkinson’s Study, a multi-level clinical, molecular and device-based initiative for defining early diagnosis and progression markers of patients with typical and atypical parkinsonism [41] (Table 1). This exploratory unbiased multi-centered designed study aims to integrate a comprehensive longitudinal clinical assessment accompanied by an omics-based molecular fingerprints analysis from a high-quality bio-samples collection, including genomics, transcriptomics and metabolomics/proteomics profiles from blood, saliva, urine, skin and CSF, integrated with a device-based assessment via the use of an open-source digital platform to harmonize international PD cohort studies. Such a multidimensional, still ongoing approach, ranging from genes and complex molecular fingerprints to the longitudinal clinical assessment, may facilitate the detection of PD subtypes and disease-specific biomarkers and precision medicine [41].

Furthermore, the integration of genome-wide association studies, together with expression and epigenetic datasets, has recently suggested that gene regulation data may be used to identify candidate the genes and genomic processes underlying the risk of sporadic PD [42] (Table 1). In particular, Kia and colleagues, using various complementary bioinformatics tools, integrated GWAS, the transcriptome-wide association study (TWAS) and methylation data and identified 11 candidate genes whose regulatory changes in expression, splicing or methylation are associated with the risk of PD. Moreover, coexpression and protein level analyses of these genes demonstrated a significant functional association with known mendelian PD genes. Future efforts in multilevel omics data integration along with advances in the understanding of PD pathogenesis will refine current classification systems and biomarkers in order to assign treatments and shape the most effective therapeutic approaches.

## 4. Amyotrophic Lateral Sclerosis

Amyotrophic lateral sclerosis (ALS) is a progressive and lethal neurodegenerative disease that affects upper and lower motor neurons, resulting in progressive muscular paralysis and death, which predominantly occurs due to respiratory insufficiency. ALS affects about 3–5 out of every 100,000 individuals worldwide, representing the most common motor neuron disease in adults [79,80]. The disease arises sporadically (SALS) in the majority of cases, while nearly 10% of patients have a family history (FALS) [81]. Currently, there is no cure or prevention for ALS, and the only licensed medications, Riluzole and Edaravone, are largely symptomatic and provide modest effects on disease progression only in some patients [82,83,84,85,86]. Many factors may have contributed to the slow progress in developing effective treatments for this devastating disease, including the complex and heterogeneous nature of ALS pathogenesis, characterized by distinct clinical features and progression patterns, together with a plurality of associated genes. In fact, since the discovery of mutations in SOD1, in 1993, as the first gene to be linked to ALS, an increasing number of causal and risk genes have been identified, revealing a high degree of genetic heterogeneity. Disease heterogeneity is also reflected by the involvement of different mechanisms in ALS pathogenesis, including mitochondrial dysfunction and oxidative stress, defective axonal transport, excitotoxicity, apoptosis, neuroinflammation, impaired DNA binding and repair and aberrant RNA-processing [87]. Within this context, it is clear that disentangling the phenotypic and genotypic heterogeneity of ALS may not only improve the comprehension of the complexity of this disease but also, above all, facilitate an appropriate stratification of ALS patients into disease subgroups for clinical research purposes.

The advent of numerous high-throughput “omics” studies in the past decade have started to provide a better understanding of the molecular basis underlying disease heterogeneity, enabling researchers to differentiate ALS from healthy controls and stratify ALS patients into distinct subgroups, paving the way to the development of efficient and effective personalized diagnostics and patient-guided therapies [88]. To this end, in the last years, our research group has established, for the first time, the foundation for a functional molecular classification of ALS, by stratifying the transcriptomes of SALS postmortem cortex samples into two distinct molecular subtypes (SALS1 and SALS2) characterized by different combinations of genes and pathways that were deregulated [43,44] (Table 1). In particular, SALS1 showed predominant signatures of aberrant extracellular matrix remodeling and antigen processing and presentation, while the largest SALS2 cluster displayed hallmarks of axonal damage, oxidative stress and neuroinflammation. Of note, a stratification of ALS patients has been reproduced in a recent study conducted by Tam et al. (2019), and other transcriptome profiling-based studies confirmed the existence of distinct molecular subtypes of ALS [45,89] (Table 1). Considering the multifactorial nature of the disease processes driving ALS pathogenesis, we explored the ALS heterogeneity at different levels of omics data, by integrating our gene expression profiling with the analysis of alternative splicing and genomic structural aberrations occurring in the same ALS patient cohort [90,91] (Table 1). In particular, we observed the differential expression of a substantial number of genes encoding splicing factors in both the motor cortex and spinal cord samples of two molecularly separated ALS subgroups, revealing a significant overexpression for SALS1 and a down-regulation for SALS2. In addition, we also characterized copy number variants occurring in these patients, identifying subtype-specific genomic alterations positively correlated with transcriptional signature profiles, suggesting that genomic and transcriptomic alterations may complement each other in driving the molecular heterogeneity underlying ALS pathogenesis [90].

In addition to genomics and transcriptomics, other innovative omics sciences, including metabolomics, have allowed the identification of specific markers and signatures that can distinguish between ALS patients and healthy individuals, as well as stratify SALS patients into different subgroups [46,47,92,93,94]. One striking example was reported in the study of Wuolikainen and colleagues, where ALS patients bearing different SOD1 mutations presented a very distinct metabolic signature (including a decrease in amino acids in the CSF) in comparison to patients without SOD1 mutations (both familial and sporadic cases), and this classification was also observed between homozygous and heterozygous carriers of these ALS genetic variants, correlating with different disease progression patterns [46]. Similarly, low urate plasma levels were related to a higher risk of developing ALS, years before the onset of symptoms [95]. In another study, researchers categorized the metabotype of skin-derived fibroblasts from SALS patients into different subgroups characterized by distinct metabotypes, one of them typified by increased trans-sulfuration pathway-derived cysteine to support GSH biosynthesis and glucose hypermetabolism, in comparison with controls and other SALS subgroups [47].

Overall, the documented patient stratification and their peculiar molecular portraits lay the foundations for developing more efficacious and individualized therapeutic interventions for ALS. In this context, our analyses provided a series of potential biomarkers and therapeutic targets differentially deregulated in specific subsets of ALS patients, suggesting their utility in the establishment of medicine based on individual molecular-level profiles [96,97]. Among these, an example is represented by histamine-related genes that we found deregulated at the genomic and transcriptomic level in the motor cortex, as well as in the spinal cord of two molecular-based subgroups of SALS patients, supporting the hypothesis that histamine-related target genes might represent candidate biomarkers and targets for patient-oriented ALS care [98]. In this regard, preliminary results indicated that the pharmacological modulation of this signaling seems to ameliorate ALS features, improving motor performance and survival in ALS mice and increasing motor neuron survival in ALS models [98,99,100].

## 5. Conclusions

The recent scientific breakthroughs and technological advancements have improved our understanding of disease pathogenesis and changed the way we diagnose and treat disease, leading to a more precise, predictable and optimized health care. The combination of deep clinical phenotyping, multi-omics technologies and advanced molecular profiling has provided benefits in several areas of medicine, especially in oncology, and there is great enthusiasm to translate these approaches to NDs as soon as possible.

The evidence documented here shows that a deeper understanding of NDs based on multi-omics levels may prompt a shift toward their molecular classification, highlighting both the intrinsic heterogeneity of the pathologies and the differences in involved molecular pathways, as well as the relationships and connections inside the neurodegenerative process itself. Gathering multi omics-layers (genomic, transcriptomic and proteomic data) from 177 studies and more than one million patients suffering AD, PD, Huntington’s disease (HD) and ALS has recently shown a remarkably high number of shared differentially expressed genes between the transcriptomic and proteomic levels for all conditions, shedding light on processes like the humoral immune response, that have previously been described only for certain diseases [101]. An accurate investigation of complex biological systems by integrating multiple underlying data sources may help to detect shared genetic patterns between the neurodegenerative diseases, identifying biomarkers for differentiating disease states and thereby facilitating the decision-making process and treatment management.

Although these great technological breakthroughs are certainly encouraging, a routine implementation of omics data on clinical decisions for personalized interventions in NDs will require further improvements in data acquisition, analysis and cost-effectiveness. In this regard, the most formidable challenges lie perhaps in data analysis and storage, as well as in elucidating the biological meaning of various omics data, differentiating causality from correlation. This aspect is further amplified when working with human brain samples, where sample size, subject variation (gender, ethnicity, age, diet and lifestyle choices), brain region, disease stage, tissue quality and technical noise can confound or obscure findings. Therefore, to make omics data concretely suitable for molecular signature discovery, the samples must be collected through appropriate and standardized experimental and analytical procedures, in order to reduce the prevalence of batch effects. If they are not carefully controlled, these factors can be incorrectly associated with the clinical phenotype of interest, leading to the development of a classifier that performs very well on the data used in its development but poorly on independent test samples. Another aspect to consider is that although the cost of omics analyses continues to decrease, an although multiple high-throughput data can guide individualized treatment regimens and be integrated into the clinic, their analysis and integration still require extensive and time-consuming human input for correct and reliable data interpretation, due to the heterogeneous and wide variability of the datasets generated. Therefore, going forward, standardized bioinformatically integrated omics approaches and better statistical methods will be required to identify and extract the correct biological meaning from omics data.

To be efficient and translatable in the clinic, the integrated whole systems approach would require coordinated collaborative efforts among the clinical, pharmaceutical and biotechnological industries, and researchers’ communities to foster new joint multidisciplinary applications that will enable the translation of findings into neurology practice. To this end, a number of major international initiatives and funding grants have been promoted by international agencies to support the development of omics-based projects fostering precision medicine (e.g., ERACoSysMed—Systems Medicine to address clinical needs; Personalised Medicine for Neurodegenerative Diseases—National Science Centre, Poland; IMI Innovative Medicines Initiative; Clinical utility of omics for better diagnosis of rare diseases by CORDIS EU research) and are still ongoing. We believe that, if successful, such collaborations could increase the power to identify new statistically significant ND-associated alterations and build more accurate prediction models, providing opportunities to the clinical implementation of stratification for treatments with the potential to transform ND management and dramatically improve patient outcomes.

## Figures and Tables

**Table 1:** Exemplary studies of omics approaches and/or their integrative analysis for stratifying NDs into their different molecular subtypes.

	Study (Year)	Sample	Omics Technique	Main Findings	Ref.
**AD**	*Nativio et al. (2020)*	Postmortem human brain samples (lateral temporal lobe, Brodmann area 21 or 20) of AD patients (*n* = 12; mean age = 68), cognitively healthy older individuals (*n* = 10; mean age = 68) and healthy younger individuals (*n* = 8, mean age = 52) obtained from the Center for Neurodegenerative Disease Research brain bank at the University of Pennsylvania.	Transcriptomics, proteomics and epigenomics	Multi-omics analysis revealed that AD involves a reconfiguration of the epigenome, wherein H3K27ac and H3K9ac affect disease pathways by dysregulating transcription and chromatin–gene feedback loops.	[28]
	*Xicota et al. (2019)*	Blood and plasma samples from 48 individuals amyloid positive and 48 amyloid negative (enrolled at the Pitié-Salpêtrière University Hospital, Paris, France).	Transcriptomics (RNA-sequencing), metabolomics and lipidomics using liquid chromatography-mass spectrometry	This study suggests a potential blood omics signature for the prediction of amyloid positivity in asymptomatic at-risk subjects.	[27]
	*Clark et al. (2020)*	Cerebrospinal fluid of 120 individuals, aged 55 or older, including subjects with normal cognition, mild cognitive impairment (MCI) or mild AD dementia were enrolled at the University Hospital of Lausanne, Switzerland.	Genetics, proteomics, metabolomics, lipidomics, one-carbon metabolism and neuroinflammation markers	Multi-omics integration identified five major dimensions of heterogenicity, explaining the variance within the cohort and differentially associated with AD. The analysis also identified combinations of a group of molecules that significantly improved the prediction of both AD and cognitive decline.	[26]
	*Ma et al. (2019)*	10,441 unrelated non-Hispanic white individuals (5522 with AD, 4919 cognitively normal controls) in the Alzheimer’s Disease Sequencing Project case-control WES data set.	Genomics (whole-exome sequencing), genome-wide association analyses	This study highlighting the possibility to stratify AD patients based on their APOE genotype. In fact, the APOE ε4 allele shows a dose-dependent relationship with increased risk for late-onset and sporadic cases of AD, while the inheritance of the ∊2 allele is protective.	[32]
	*Dagan et al. (2020)*	951 brain samples, obtained from up to 17 brain regions of 85 AD patients with varying severities of AD neuropathology and 22 non-demented subjects. All subjects ranged from 60 to 100 years of age.	Transcriptomics (Expression array)	The authors identified different altered transcriptional signatures characterized AD samples vs non-demented samples and specific transcriptional signatures associated with different subsets of AD patients, demonstrating the high molecular variability and complexity of gene expression in AD.	[33]
	*Milind et al. (2020)*	Post-mortem brain from 2114 human samples from three cohorts of patients with late-onset AD (including 312 North American Caucasian patients and 987 individuals from across the United States).	Genomics (whole-genome sequencing), transcriptomics (RNA-Sequencing)	The authors identified different molecular subtypes of late-onset AD patients associated with specific biological pathways and molecular processes.	[34]
	*Neff et al. (2021)*	1543 transcriptomes across five brain regions in two AD cohorts (the Mount Sinai/JJ Peters VA Medical Center Brain Bank (MSBB-AD) and the Religious Orders Study–Memory and Aging Project).	Transcriptomics (RNA-Sequencing)	The authors identified three major molecular subtypes of AD corresponding to different combinations of multiple dysregulated pathways and subtype-specific drivers.	[35]
	*Iqbal et al. (2005)*	CSF samples of 468 clinically diagnosed Finnish and Swedish Alzheimer’s disease patients (N = 353) or non-Alzheimer’s subjects (N = 115) (mean age = 70)	Proteomics	The authors identified five AD subgroups based on CSF levels of Aβ1-42, tau, and ubiquitin; each subgroup presented a different clinical profile.	[36]
	*Toschi et al. (2019)*	CSF samples from 113 participants (20 healthy controls, 36 subjective memory complainers, 20 mild cognitive impairment, and 37 AD dementia). The multicenter cross-sectional study includes subjects from France, Germany and Sweden. All subjects ranged from 60 to 77 years of age.	Proteomics	The authors found a set of biologically defined clusters not significantly linked to the clinical diagnosis but exclusively based on core biological fluid markers which reflect distinct pathomechanistic alterations associated with the disease (i.e., brain Ab accumulation and neurofibrillary pathology, neuro-inflammation, axonal damage, and neurodegeneration).	[37]
PD	*Lerche et al. (2021)*	CSF samples from 516 PD patients (102 PDGBA, 414 PDGBA_wildtype). The multicenter cross-sectional study includes subjects from United States, Europe, Israel, and Australia.	Genetics, proteomics, metabolomics	The authors demonstrated that variants in the glucocerebrosidase gene (GBA) may allow patient stratification for clinical trials merely based on mutation status and that might serve as a biochemical read-out for target engagement.	[38]
	*Prasuhn et al. (2019)*	The study is ongoing. So far, >950 PD patients have been included.	Genomics, genome-wide association study	This study focuses on genetically stratified subgroups of Parkinson’s disease patients (PD) with enrichment of risk variants in mitochondrial genes, assuming that individuals with a “higher mitochondrial burden” will likely respond to coenzyme Q10.	[39]
	*Carling et al. (2020)*	Skin fibroblasts of 100 sporadic PD patients (sPD) and 50 age-matched controls (age in years ± standard deviation (SD): sPD patients 61 ± 10.7 years; controls, 61 ± 13.1 years) from the Oxford Parkinson’s Disease Centre Discovery cohort and Sheffield Teaching Hospitals in UK.	Transcriptomics (RNA-sequencing), genomics, proteomics.	The authors identified distinct subgroups with mitochondrial (mito-sPD) or lysosomal (lyso-sPD) dysfunctions, sustaining the utility of using skin fibroblasts to undertake mechanistically rather than clinically defined sPD subgroups.	[40]
	*Hipp et al. (2018)*	The study is ongoing. So far, 498 patients and 520 healthy control have been included. The study includes all patients with parkinsonism in Luxembourg and the surrounding ‘Greater Region’ (including the German, French, and Belgian border regions).	Genomics, genotyping, transcriptomics, metabolomics/proteomics	The authors envision the Luxembourg Parkinson’s study as an important research platform for defining early diagnosis and progression markers that translate into stratified treatment approaches. The study is ongoing.	[41]
	*Kia et al. (2021)*	GWAS: 26,035 PD patients and 403,190 controls of European ancestry; eQTL Data: 134 control individuals (frontal cortex, temporal cortex, occipital cortex, hippocampus, thalamus, putamen, substantia nigra, medulla, cerebellum, and white matter); genome-wide methylation: substantia nigra and the frontal cortex of 134 individuals with PD from the Parkinson Disease UK Brain Bank.	Genome-wide association study, genomics, transcriptomics, epigenomics	The authors identified candidate genes whose change in expression, splicing or methylation are associated with the risk of PD. Interaction network analyses also highlighted the functional pathways and cell types in which these candidate genes have an important role.	[42]
ALS	*Aronica et al. (2015); Morello et al. (2019)*	Post-mortem motor cortex from caucasian SALS patients (31, mean patient age = 57)) and control individuals (10, mean patient age = 55 years).	Transcriptomics (gene expression array), genomics	The authors demonstrated the utility of an integrative multi-omics molecular classification of ALS, by stratifying the genomes and transcriptomes of SALS postmortem cortex samples into two distinct molecular subtypes (sALS1 and sALS2) characterized by different combinations of genes and pathways.	[43,44]
	*Tam et al. (2019)*	Frontal cortex samples from 77 ALS patients and 18 neurological and non-neurological controls from the NYGC ALS Consortium.	Transcriptomics (RNA-sequencing), genomics, proteomics	Unbiased machine learning algorithms identified three distinct ALS patient molecular subtypes representing both ALS disease-implicated signatures as well as additional correlated pathways.	[45]
	*Wuolikainen et al. (2012)*	Cerebrospinal fluid (CSF) from 16 ALS patients with 6 different mutations in the *SOD1* gene compared with ALS-patients without mutations	Metabolomics (GC-TOFMS platform)	The authors found that patients with SOD1 mutations have a distinct metabolic profile in CSF and highlight the utility of metabolomics signature to distinguish ALS entity	[46]
	*Chen et al. (2018)*	77 ALS -derived dermal fibroblast lines and 45 age/sex-matched controls.	Metabolomics (LC-QTOF platform)	The authors emphasize that sporadic ALS patients can be stratified into metabotypes, helping future development of personalized medicine.	[47]

## Data Availability

Not applicable.
